# Expandable Intravertebral Implants: A Narrative Review on the Concept, Biomechanics, and Outcomes in Traumatology

**DOI:** 10.7759/cureus.17795

**Published:** 2021-09-07

**Authors:** Diogo L Moura, Josue P Gabriel

**Affiliations:** 1 Spine Surgery, Spine Unit, Orthopedics Department, Coimbra Hospital and University Center, Coimbra, PRT; 2 Spine Surgery, Spine Institute of Ohio, Grant Medical Center, Columbus, USA; 3 Orthopedic Spine Surgery, Spine Institute of Ohio, Grant Medical Center, Columbus, USA

**Keywords:** expandable intravertebral implants, fractures, vertebral, compression, restoration, reduction, anatomical, endplates

## Abstract

Expandable intravertebral implants are self-expanding devices applied percutaneously by the posterior transpedicular approach. These devices introduce the concept of anatomical restoration of vertebral body endplates and direct anatomical reduction performed from the interior of the vertebral body with a compression fracture. This paper aims to provide a narrative review on the concept, indications, biomechanical characteristics, as well as functional and radiographic outcomes of the main expandable intravertebral implants currently available, in terms of their application to thoracolumbar spine traumatology. To this end, we performed a search in July 2021 on the MEDLINE/PubMed platform with the words “expandable intravertebral implant”, “armed kyphoplasty”, “Vertebral Body Stenting” or “stentoplasty” and “SpineJack”. The search yielded 144 papers, and of those, we included 15 in this review. We concluded that percutaneous transpedicular posterior access, the ability to reduce vertebral body fractures, particularly of the vertebral endplates and to maintain the vertebral body height, makes the application of expandable intravertebral implants an attractive option in the treatment of thoracolumbar vertebral compression fractures. However, more prospective, randomized, and large-scale blinded studies are still warranted, especially comparative studies between treatments and about the preferential use of an expansive implant over others, in order to gain definitive insights into the effectiveness and indications of each of these devices.

## Introduction and background

The treatment of spine fractures, particularly vertebral body compression fractures, has evolved rapidly over the last 30 years, resulting in considerable changes in indications, techniques, and surgical stents. The morbidity of anterior approaches for anterior column reconstruction has led to an exaggerated tendency to treat vertebral compression fractures by pedicle fixation, often increasing the number of fixed levels. However, it is known that the loss of support in the anterior column, a region that receives 80% of axial loads, will inevitably overload the posterior instrumentation, sometimes resulting in its failure, loss of vertebral body height, local and segmental kyphosis post-traumatic, with clinical and functional repercussions [1-4]. In light of this, minimally invasive techniques for augmenting the fractured vertebral body have gained increasing popularity due to their ability to stabilize the anterior column through a posterior percutaneous approach, allowing for good results in symptomatic relief, in convalescence speed, in functional and life quality indexes, as well as in the restoration of the anatomy and biomechanics of the spine [5-11].

## Review

Materials and methods

**Figure 1:**
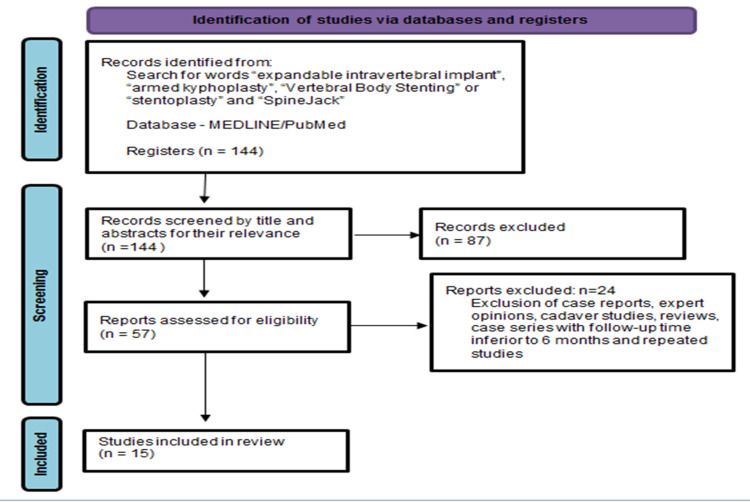
PRISMA flow diagram* *[12] PRISMA: Preferred Reporting Items for Systematic Reviews and Meta-Analyses

This paper aims to carry out a narrative review of the concept, indications in traumatology, biomechanical characteristics, as well as functional and radiographic outcomes of the main expandable intravertebral implants currently available, applied to thoracolumbar spine traumatology. To this end, we performed a literature search in July 2021 on the MEDLINE/PubMed platform with the words “expandable intravertebral implant”, “armed kyphoplasty”, “Vertebral Body Stenting” or “stentoplasty” and “SpineJack”. We initially found 144 papers, of which, after reviewing their titles and abstracts, 57 were selected because they focused on expandable intravertebral implants and their role in fractures of the thoracolumbar spine. In the next stage of the selection process, we excluded case reports, expert opinions, cadaver studies, reviews, case series with a follow-up duration of less than six months, and repeated studies, ultimately yielding a total of 15 papers for the final analysis (seven about VBS® and eight about SpineJack®) (Figure 1), which consisted of case series and comparative studies of retrospective and prospective nature.

Origin and concept of expandable intravertebral implants

Kyphoplasty emerged as an evolution of vertebroplasty, allowing to combine its analgesic and stabilizing effect concerning the application of intravertebral cement, with the restoration of the fractured vertebral body’s height, by creating an intrasomatic cavity with an expansive balloon, a space that is then filled with cement. In addition to the advantages of reducing the fractured vertebral body, the creation of a previous intrasomatic cavity with less pressure and supposedly covered by impacted bone trabeculae and by the walls of the vertebral body, which is filled with cement, reduces the risk of its extravasation, thereby allowing to minimize the risks of complications from this extravasation [5,9,13-15]. Nevertheless, one of the criticisms against kyphoplasty is the inability to maintain the restored height of the vertebral body after balloon removal and before applying the cement, resulting in vertebra flattening through elastic recoil by ligament and annulotaxis. Even with the patient's positioning on the table with the spine in hyperextension, compression forces of approximately 110 Newtons continue to act on the fractured vertebra, contributing to its flattening [4-6,13-22].

Expandable intravertebral implants are devices with self-expansion capability applied by a posterior transpedicular approach, percutaneously, inside the fractured vertebral body, usually with a compression fracture. Their expansion allows for the reduction of the fractured vertebral body, restoring its height, integrity, and stability when filled or stabilized with cement or bone graft. The application of expandable intravertebral implants, also known as armed kyphoplasty, in addition to allowing for the aforementioned analgesia and stabilization benefits of vertebroplasty and kyphoplasty, also theoretically enables the maintenance of restored vertebral height in the long term. This is made possible because the vertebral endplates, after their reduction, stay mechanically supported by the expanded device (they work as an interior support), which decreases or prevents vertebral flattening after expansion, reducing the risk of posttraumatic local and segmental kyphosis, and ensuring stable anterior column support at the level of the vertebral body [5,6,13,15,23-26].

Classically, kyphoplasty, with or without expandable intravertebral implants, was indicated for acute compression fractures of the vertebral body of osteoporotic or metastatic origin. However, the excellent results obtained have led surgeons to extend their indications to traumatic fractures in patients of both advanced and young ages. The value of flattening and kyphosis of the vertebral body that justify its reduction is not well-defined in the literature. Nonetheless, some authors point to a flattening of about one-third of the vertebral body’s height, vertebral kyphosis equal to or greater than 15°, and/or Beck Index less than or equal to 0.7 [15,23,27-29]. It is increasingly considered that the reconstruction of the anterior column, in particular of the vertebral body, which is an important support for axial loads predominant in bipedal gait, is essential to reconstruct a spine that is biomechanically and physiologically closer to that prior to the fracture [6,30,31]. Therefore, it is currently considered that the application of expandable intravertebral implants is indicated for compression fractures of the vertebral body, that is, type A1, A2, A3, or A4 fractures of the AO Spine classification; however, it should be noted that there is a chance for conservative treatment, particularly in type A1, A2, and A3 fractures [15,28,32,33]. Conservative treatment may be an option when patients are able to upright the trunk without relevant pain. However, pain relief, getting up and walking, as well as the rest of the recovery, are usually faster in operated patients (getting up and walking in a few hours and unrestricted activity in 24 hours, often without any pain when stabilizing with cement), in addition to obtaining a more adequate reduction of the vertebral body with the surgical solution of expandable intravertebral implants application [9,14,15,19,20,33-38].

Relevance of anatomical reduction in vertebral compression fractures

Several authors currently draw attention to compression fractures of the vertebral bodies, addressing the need to obtain an anatomical restoration or the closest one (vertebral kyphosis angle, vertebral height, and morphology of the vertebral endplates), similar to the goal in other joints of the human body [5,14,19,20,28,39-42]. The theoretical objectives of this anatomical restoration are presented in Table 1.

**Table 1:** Anatomic reduction goals in compression vertebral body fractures* *[5,14,19,20,28,39-42]

Patient context	Goals
Resistant bone: young age	Original anatomic restoration of vertebral endplates allows recreating the original position of the frequently injured intervertebral disc, which promotes its proper healing, pressurization, and nutrition. This theoretically allows a more physiological load-dampening function of the intervertebral disc and potentially minimizes its degeneration. In this way, a biomechanically and functionally more similar spine to the one previous to the fracture is guaranteed, restoring its sagittal balance, searching to minimize the progression of the disc, and osteodegenerative changes of that vertebral segment and the adjacent levels
Porotic bone: advanced age	The vertebral body height restoration at the first osteoporotic vertebral fracture is essential to prevent the domino effect of osteoporotic spine disease, which is the consecutive occurrence of osteoporotic fractures in adjacent vertebrae due to anterior column overload after the first uncorrected vertebral wedging. Wedging the vertebrae progressively shifts the load axis to a more anterior position, exposing the osteoporotic vertebral bodies to excessive loads, favoring kyphotization of the spine

**Figure 2:**
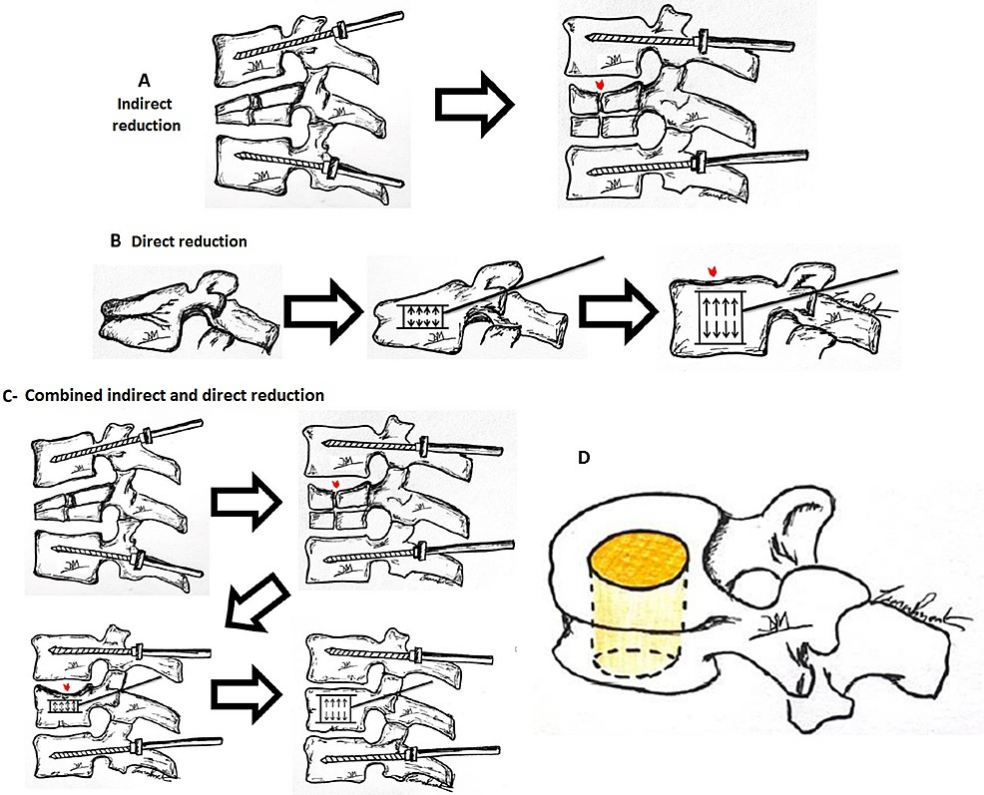
Indirect and direct fracture reduction A: Indirect fracture reduction through distraction and lordosis maneuvers performed by pedicular instrumentation of adjacent vertebrae. Note the reduction of posterior wall retropulsion, as well as the restoration of anterior and posterior sagittal heights of the vertebral body. However, a central sinking of the superior vertebral endplate persists, with no complete restoration of the mid-sagittal height of the vertebral body (red arrowhead). B: Direct reduction of the fracture by intravertebral expandable implants. Note the elevation of the entire upper vertebral endplate (red arrowhead). C: Combined indirect and direct reduction methods. Note the complementarity of indirect reduction with direct reduction. Direct reduction by intravertebral expandable implants is the only way to obtain a reduction in the central region of the vertebral endplates. D: Endplates central region – reduction only possible by direct methods with intravertebral expandable implants

Expandable intravertebral implants introduce the concept of direct fracture reduction, that is, performed by an implant expanded at the exact same location of the fracture inside the vertebral body. If the fracture is caused by a compression mechanism, these implants will do the opposite, expanding the vertebral body, which is the opposite mechanism to the one that caused the fracture, and hence a very effective method of fracture reduction. The classic indirect reduction by distraction and lordosis maneuvers, through pedicular instrumentation of the adjacent vertebrae, reduces, above all, the cortical ring of the vertebral body, due to the containment effects of the anterior and posterior longitudinal ligaments, and the peripheral portions of the vertebral endplates by containment of the fibrous annulus of the intervertebral disc. In turn, only direct reduction using expandable intravertebral implants allows restoring the central portion of the vertebral endplates, as presented by Baeesa et al., in a study involving three-dimensional CT reconstruction of vertebrae after the application of expandable intravertebral implants, which demonstrates their importance in anatomical reduction and favoring adequate disc healing in a post-traumatic context (Figure 2). Furthermore, these expandable implants, given the integrity of the anterior and posterior common longitudinal ligaments, as well as the insertion of the fibrous annulus in the vertebral endplates, also allow anterior and posterior bone fragments to effectively return to their original position. Thus, they also reduce the peripheral portion of vertebral endplates and the cortical ring itself [15,17,28,43-46].

In short, several authors consider that to obtain the desired complete anatomical reduction of a compression vertebral body fracture, direct reduction with expandable intravertebral implants is mandatory in order to correct the central depression of the vertebral endplates. In some fractures, this maneuver is sufficient for total fracture reduction, while in others, it may be necessary to associate adjacent pedicle instrumentation to perform indirect reduction maneuvers [2,5,27,33,43,44,46-48].

Types of expandable intravertebral implants and their biomechanics

In Table 2, we present the characteristics of the two main expandable intravertebral implants currently available: Vertebral Body Stenting (VBS®) and SpineJack® systems, which are the expandable intravertebral devices most commonly applied worldwide nowadays. The authors chose to focus only on these two expandable intravertebral implants, which are the most used ones and about which the most extensive scientific literature is available; however, it is important to keep in mind that there are other similar devices, albeit with less robust scientific evidence.

**Table 2:** Characteristics of the two main expansive intravertebral implants* *[6,19,21,26,33,42,47]

Implant designation	VBS® (Vertebral Body Stenting)	SpineJack®
Morphology	Cylindrical shaped mesh (stent), 2 implants by transpedicular access	Similar to a car jack, with superior and inferior lamellas, 2 implants by transpedicular access
Material	Chromium-cobalt	Titanium
Expansion direction	Centrifugal circumferential in the coronal plane (craniocaudal + lateral)	Bidirectional in craniocaudal or vertical direction
Expansion mechanism	Hydraulic mechanism, through a kyphoplasty balloon (controlled pressure and volume)	Mechanical mechanism
Expansion power	Maximum pressure = 30 Atm; Maximum expansion volumes: #small stent = 4 mL; #medium stent = 4.5 mL; #large stent = 5 mL	Expansion force = 500 Newtons; maximum expansion heights: #small implant 4.2 = 12.5 mm; #medium implant 5.0 = 17 mm; #large implant 5.8 = 20 mm
Objective	Fracture reduction and space occupation – indication in osteopenia, lytic injuries, and A4 burst traumatic fractures	Fracture reduction, preservation of unfractured trabeculae – indication in A1, A2, and A3 fractures with healthy bone
Rationale	VBS® is a reducing and space-occupying implant, since it has a multidirectional expansion (vertical and lateral). It is indicated for reconstruction or replacement of the vertebral body, not intending to wait for vertebral fracture healing. Stents are implants that, due to their expansion and impaction of the surrounding bone trabeculae, form two cavities inside the vertebral body covered by an envelope of impacted trabeculae. These implants form cavities that, after being filled with cement or bone graft, replace a large part of the vertebral body, filling it and stabilizing it. In addition, they minimize cement leakage by recreating the vertebral body walls by impacting bone trabeculae, thereby containing the cement inside	SpineJack® is a more powerful reduction implant and preserver of unfractured native trabeculae. This implant is not a space occupant as it has only vertical and not lateral expansion. In these cases, the goal is to reduce the fracture and wait for its healing, rather than replacing the vertebral body. This implant only reduces and supports the vertebral body, as it does not have a cavity shape or lateral expansion; it does not destroy intact lateral trabeculae and does not create significant empty space within the vertebral body. As such, it is useful if we want to reduce the fracture and obtain bone healing, preserving as much healthy bone as possible. We consider this implant not ideal for replacing the comminuted, lytic, or porotic vertebral body, which has no interior stable content and needs intrasomatic filling in addition to fracture reduction

In summary and according to the table, VBS® reduces and replaces the flattened and destroyed vertebral body, while SpineJack® reduces and preserves the flattened vertebral body. As such and based on literature review and device biomechanics, authors tend to prefer, according to the AO Spine classification, the SpineJack® for type A1, A2, and A3 fractures in healthy bone, reserving the VBS® for type A4 fractures in the healthy bone (associated with pedicular fixation at adjacent levels) and for compression vertebral body fractures in the porotic bone.

The reduction quality is totally dependent on the positioning of the intravertebral implants in the vertebral body before their expansion, and this positioning must be adequate to the fracture pattern and to the intended degree of reduction. This step is crucial since an incorrect positioning of the implants can not only prevent their proper expansion and reduction but can even invade the vertebral body cortical walls, with the neurological and even vascular risks that may be associated with it [5,48]. It is also important to bear in mind that once the expansion of these current devices is started, it is irreversible, and it is not possible to decrease its size or to remove the expanded implants in the same percutaneous way used for its insertion [47,48]. VBS® stents (stentoplasty) were adapted from the principle of vascular stents to the spine. The expansion of the metallic mesh of these implants creates intrasomatic cavities by the impaction of vertebral body trabeculae. They have the advantage of creating these low-pressure areas, contained by the implant mesh surface and the impacted surrounding trabeculae, which, in theory, lowers the risk of cement leakage compared to vertebroplasty, kyphoplasty, and even other intravertebral implants that do not have this cylindrical shape [5,6,26,29,43,49]. However, the cavitary intrasomatic filling with cement and its containment by the cylindrical implant reduces its interdigitation in the bone trabeculae, which, in theory, can reduce the stability of the stents inside the vertebral body, and there may be a higher risk of their migration [5]. Still, the metallic mesh reinforced with the cement simulates the concept of reinforced concrete from civil construction, allowing for a stable and resistant reconstruction of the vertebral body. Furthermore, the expansion of the balloon enclosed by the stent ensures a uniform and more predictable cylindrical expansion than the balloon alone in kyphoplasty. In fact, the balloon alone, as it is not a rigid structure, is more likely to insinuate itself into areas of lower pressure and create an anomalous cavity, being less predictable in reducing the vertebral body [26,49].

One of the points in favor of SpineJack® is the fact that, due to its smaller size compared to, for example, VBS®, and its only vertical expansion mechanism, it does not create cavities inside the vertebral body, only impacting the minimum amount of bone trabeculae needed to reduce compression fractures, that is, those above and below the implants. During the implant expansion, two vertical cracks are created, which are then filled with cement, resembling two vertical parasagittal pillars supporting the vertebral endplates. The injected cement often interdigitates in the bone trabeculae between the two implants, joining them in a bridge and creating a sort of supporting ring of the vertebral endplates, which allows to increase the support areas and better distribute vertebral loads. In this way, SpineJack® allows preserving more healthy bone trabeculae than the multidirectional expansion mechanism, which is useful to ensure greater cement interdigitation in the preserved trabeculae and, consequently, a larger cement-trabeculae contact area. This increases the stability of the construction and the implants inside the vertebral body while requiring less cement to stabilize the devices [15,20,47,50]. Moreover, preserved trabeculae may be particularly important at young ages, maintaining a biomechanical structure closer to the original vertebral body compared to a body with big cavities filled with cement or bone graft. Also, the smaller amount of cement needed to stabilize these implants reduces the probability of its extravasation and is beneficial as it does not increase vertebral rigidity as much, theoretically decreasing fractures at adjacent vertebras [50-52]. SpineJack® mechanical expansion mechanism makes it possible to overcome superior bone strengths, compared to the VBS® hydraulic mechanism, as the latter is limited by a maximum pressure value from which the implant does not expand, while the limit of SpineJack® is only its maximum size.

Radiographic and functional outcomes of fractured vertebral body reconstruction with expandable intravertebral implants

We present the main current studies regarding the application of expandable intravertebral implants in the context of compression vertebral fractures (Table 3, 4).

Klezl et al. were pioneers in exploring concerns with regard to the use of VBS® stents in traumatic compression fractures in the thoracolumbar spine [5]. The stents were applied to type A1.3 and A3.1 fractures of the AO Spine classification and to osteoporotic fractures, in a total of 20 fractures. With a mean follow-up of one year, they found a significant improvement in the visual analog pain scale (VAS) from 9.7 at baseline in the traumatic group to 2.7 at six weeks postoperatively, 2.2 at six months, and 1.6 after one year. In the osteoporotic group, it went from 8.9 initially to 4.8 at six weeks, 4 at six months, and 2.5 after one year. Regarding the Oswestry Disability Index obtained, it was 20.4% (6-33%) in the traumatic group and 41.7% (14-58%) in the osteoporotic group. The mean reductions in vertebral body angulation obtained were 7.3⁰ in the traumatic group (vertebral angle went from 13⁰ to 5.7⁰) and 4.5⁰ in the osteoporotic group (vertebral angle went from 9.7⁰ to 5.2⁰), with no loss of reduction during the time of follow-up. There were two cases of cement leakage without any clinical implications [5]. In the same year, Muto et al. described the use of VBS® for the treatment of osteoporotic and traumatic vertebral fractures with 12 months of follow-up [18]. The height in the fractured vertebral body was increased in 12 of the 20 vertebrae by an average of 1.5 mm. No vascular, extraforaminal, or epidural leakage, or other adverse events were observed. The authors recorded a reduction of four scores in the VAS evaluation and a 40% reduction in the ODS score compared with the pre-treatment values. They concluded that the mechanical scaffold of the stent restores and maintains the height and at the same time offers a cavity for injection of highly viscous polymethylmethacrylate (PMMA) cement without increasing the rate of adjacent vertebral fractures [18].

Hartmann et al. retrospectively studied 18 incomplete explosive traumatic thoracolumbar fractures submitted to the application of VBS® stents [40]. Vertebral kyphosis had an average improvement of 3.2⁰ (10.4⁰ to 7.2⁰), while segmental kyphosis showed an improvement of 5⁰ (9.9⁰ to 4.9⁰). In turn, Beck Index went up from 0.79 to 0.89. However, there was a loss in corrections of vertebral and segmental kyphosis over the two years of follow-up, of 0.8⁰ and 2.1⁰ respectively. Mean VAS improved from 8 at baseline to 4 postoperatively and then to 2 at the end of follow-up. Functional scores after two years of meantime were mean Oswestry Disability Index of 28.9% and mean SF-36 of 61.1%, corresponding to moderate limitation of activities of daily living and quality of life. There was cement leakage in two cases, without clinical repercussions [40]. The study by Thaler et al. verified in 55 osteoporotic vertebral fractures, concerning 27 patients submitted to armed kyphoplasty with VBS®, a mean segmental kyphosis correction of 5.8°, and a vertebral kyphosis correction of 3.5° [48]. They found an improvement in the anterior-medial-posterior heights of the vertebral body of 3.6-7.3-2.2 mm, which corresponded to an improvement in the sagittal index from 0.87 to 0.94 in the VBS® group, results that were significantly superior to those of a control group of patients undergoing vertebroplasty. On the one hand, the authors detected, by CT, 25.5% of cement leakage situations in the stent group, compared to 42.1% in the vertebroplasty group, all asymptomatic. In the stent group, three fractures in the adjacent vertebrae were recorded in two patients [48]. On the other hand, in a multicenter study by Diel et al. in 100 patients (62 with osteoporosis) and 103 type A1 and A3.1 compression fractures treated with VBS®, there was an average 4.2⁰ correction of vertebral kyphosis, increasing from an average local kyphosis of 13.1⁰ to 8.9⁰.26 [27]. The anterior-medial-posterior heights of the vertebral body went up from 20.3-17.6-28.0 mm preoperatively to 24.5-24.6-30.4 mm postoperatively, which corresponded to an improvement in the Beck Index, from 0.73 to 0.81. They found a rate of 29.1% of cement leakage, with only one case being symptomatic. During the first three months of follow-up, they recorded 9% of adjacent fractures, all in the osteoporotic group [27].

Schützenberger et al., in a retrospective study of 49 patients with osteoporotic compression fractures, compared the application of VBS® with kyphoplasty, both filled with calcium phosphate biological cement, and identified a significantly lower loss of vertebral height in the VBS® group throughout the mean follow-up period of 3.75 years (local kyphosis angle loss of 7.5⁰ ±4.8 in VBS® vs 10⁰ ±5.3 in kyphoplasty and Cobb angle loss of 6.5⁰ ±8 in VBS® vs 15.4⁰ ±11 in kyphoplasty) [53]. However, there were no differences in the capacity of initial vertebral reduction, in terms of clinical (VAS 2.0 ±2.3 vs 2.2 ±2.5) and functional (Oswestry 16.6 ±17.6 vs 16.7 ±19.7) parameters and in cement leakage rates (44% vs 23%) [53]. Garnon et al., in a retrospective case series of traumatic non-osteoporotic fractures treated with VBS®, found mean vertebral height gain, vertebral kyphosis angle correction, and Beck Index improvement of 3.8 mm, 4.3°, and 0.07, respectively [54]. Stents recoil following balloon removal was observed in 47% of cases (8% ‘‘major’’; 39% ‘‘minor’’), with ‘‘major’’ recoil characterized by a loss of vertebral height gain of more than 2 mm. However, the authors highlight that despite these numbers, VBS significantly reduces vertebral body recoil compared with kyphoplasty, in which there is no load-bearing device within the cavity created after balloon deflation and before cement injection [54].

Distefano et al. applied the stent-screw-assisted internal fixation to reduce vertebral compression fractures, reconstruct the vertebral body, and fix it to the posterior elements by a technique previously described by the same group [49,55]. The technique rationale is that the stents obtain and maintain fracture reduction while the pedicle screws anchor the VBS-cement complex to the posterior elements, avoiding its displacement, and act as a bridge across the middle column, preserving its integrity from possible collapse and splitting [49]. They treated 80 severe vertebral compression fractures, characterized by advanced collapse (Genant grade 3), a high degree of osseous fragmentation (McCormack grade 2 and 3), burst morphology with middle-column injury, pediculo-somatic junction fracture, and/or large osteonecrotic cleft with this technique. VAS scores improved with a statistically significant difference from a median of 8 in preoperative to 3 at the first month of follow-up and to 2 at six months. The final Patients' Global Impression of Change (PGIC) Scale was 5.6 ±0.9 at one month and 6.1 ±0.9 at six months, indicating a very positive patients' subjective global clinical impact. Vertebral body reconstruction was evaluated by two external experts and considered satisfactory in 98.8% of levels, based on scores regarding correct placement and expansion of the implants, cement filling, and vertebral body height restoration. Osseous subsidence around the VBS-cement complex was observed during follow-up in 20% of the cases, with mild to moderate secondary vertebral body height loss, without the onset of new symptoms, and no re-treatment or surgical intervention was needed. There was a 17.5% rate of adjacent vertebral fractures, most of them treated by vertebroplasty or stent-screw-assisted internal fixation (SAIF). Cement leakage was detected in 10% of cases on postprocedure CT, with an epidural or foraminal location in 3.8%, without clinical relevance. The authors concluded that the SAIF technique is a feasible, safe, and effective minimally invasive procedure of internal stabilization for severe osteoporotic vertebral compression fractures with middle column involvement [55].

**Table 3:** Main current studies regarding the application of VBS® implants in the context of compression vertebral fractures VAS: visual analog scale; OSW: Oswestry Disability score; VA: vertebral body angle; SA: segmental angle, PMMA: polymethylmethacrylate; SAIF: stent-screw-assisted internal fixation

VBS® article	Study type	Fracture type	Number of fractured vertebrae	Mean follow-up period	Symptoms (VAS)	Function	Imaging	Complications	Conclusion
Klezl et al. (2011) [5]	Case series, prospective	A1.3 and A3.1 traumatic and osteoporotic	20	1 year	Traumatic group: VAS 9.7 à 1.6; osteoporotic group: VAS 8.9 à 2.5	Traumatic group: OSW reduction of 20.4; osteoporotic group: OSW reduction of 41.7	Traumatic group: VA 13°à5.7°; osteoporotic group: VA 9.7°à5.2°	10% PMMA leakages	Satisfactory improvement, anterior spinal column, especially the fragmented superior endplate is nicely reconstructed
Muto et al. (2011) [18]	Case series, prospective	Traumatic and osteoporotic compression	20: 4 trauma A1; 16 osteoporotic	1 year	Reduction of four scores in the VAS evaluation	40% reduction in the ODS	60% had vertebral height increased by an average of 1.5 mm	1 stent did not expand in fracture with more than 4 weeks	VBS system reduces the collapsed vertebral body and offers good height restoration. The mechanical scaffold of the stent restores the height and at the same time offers a cavity for injection of highly viscous PMMA
Diel et al. (2013)[27]	Randomized controlled trial	A1 and A3.1 traumatic and osteoporotic	103	6 months	-	-	VA 13.1°à8.9°; anterior-central-posterior heights improvement of 4.2-7-2.4 mm	29.1% PMMA leakages, 9% adjacent fractures	VBS® showed its strengths especially in the realignment of crush and biconcave fractures
Hartmann et al. (2015) [40]	Case series, retrospective	A3 traumatic	18	2 years	VAS 8à2	Final OSW: 28.9; final SF36: 61.1	VA: 10.4°à7.2°; SA: 9.9°à4.9°	11.1% PMMA leakages, VA loss of 0.8, SA loss of 2.1	Clinical outcomes comparable with kyphoplasty. The stent allows a reconstruction of the anterior column with reduced subsequent loss of correction
Schützenberger et al. (2018) [53]	Comparative case series, retrospective (versus kyphoplasty, both with calcium phosphate cement)	Osteoporotic compression	36 vertebrae in the VBS® group	3.75 years	Final VAS 2	Final OSW: 16.6	VA improvement by 7.5°; SA improvement by 6.5°	44% cement leakages	VBS® facilitated significantly better correction of the Cobb angle in comparison with kyphoplasty. The superior radiological results achieved in the VBS group are not reflected in the clinical results
Garnon et al. (2019) [54]	Case series, retrospective	Traumatic compression A1.1, A1.2, A1.3, and A3.1	39	1 year	-	-	Vertebral height: 18.3 -> 22.1; VA: 10° -> 6.7°; Beck Index: 0.79 -> 0.86	47% stent recoil following balloon removal: 8% ‘‘major’ (with loss of vertebral height gain >2 mm) and 39% minor; 18% PMMA leakages	VBS® can significantly restore vertebral height in young patients with traumatic vertebral compression fractures
Distefano et al. (2021) [55]	Case series, retrospective	Osteoporotic compression with advanced collapse (Genant grade 3), a high degree of osseous fragmentation (McCormack grade 2 and 3), burst morphology with middle-column injury, pediculo-somatic junction fracture, and/or large osteonecrotic cleft. Treated by “stent-screw-assisted internal fixation” (SAIF) technique	80	6 months	VAS 8à2	Final Patients' Global Impression of Change (PGIC) scale: ->6.1	Vertebral body reconstruction scores (based on correct placement and expansion of the implants, cement filling, and vertebral body height restoration) assigned by the two readers were excellent at 73/80 (91.25%), good at 6/80 (7.5%), fair at 1/80 (1.25%)	10% PMMA leakages, 17.5% adjacent fractures, 20% osseous subsidence around the VBS-cement complex with mild to moderate secondary vertebral body height loss	SAIF is a minimally invasive, safe, and effective treatment for patients with severe osteoporotic VCFs with MC involvement

As for SpineJack®, a pioneering comparative randomized prospective study in 300 patients with osteoporotic compression fractures type A1 of the AO Spine classification demonstrated that the group that underwent the application of SpineJack® obtained a significantly higher restoration of the vertebral body height than kyphoplasty, with both clinical and functional outcomes similar between the two techniques [19]. The authors classified the vertebral height restoration into grade 0 (no change), grade 1 (restoration of less than 50%), and grade 2 (restoration greater than 50%), having verified grade 2 reductions in 85% of cases in the SpineJack® group, grade 1 at 12%, and grade 0 at 3%. In turn, kyphoplasty allowed grade 2 reductions in 58%, grade 1 in 26%, and grade 0 in 16%. In addition, the SpineJack® group showed significantly shorter surgical duration (40 vs 45 minutes) and required significantly less cement (4 vs 5 mL) compared to kyphoplasty. The cement leak rate in kyphoplasty was 13.33%, all asymptomatic, while there were no leaks or implant failures in the SpineJack® group. There were no cases of fractures at adjacent levels in any group, which the authors attribute to the restoration of vertebral height, preventing the domino effect of the first vertebral body flattening in the osteoporotic spine due to anterior displacement of the load axis [19].

Noriega et al., in a prospective comparative study on osteoporotic compression fractures between SpineJack® and kyphoplasty in 30 patients, identified significantly higher clinical and functional indices in the implant group at the three-year follow-up, namely: VAS (14.4 ±7.2 vs 25 ±9), analgesic consumption (28.6 vs 50%), Oswestry score (6 ±3.7 vs 10.5 ±5.4), mean EQ-5D index (0.93 ±0.11 vs 0.81 ±0.09) [56]. The group with the expandable implants still presented significantly higher values in the restoration of anterior vertebral height (10% ±13 vs 2% ±8) and central height (10% ±11 vs 3% ±7 at three years postoperatively), as well as the correction of vertebral kyphosis (-5.0° ±5.1 vs 0.4° ±3.4) and Cobb angle (−2.5° ±4.4 vs no change). The efficacy of SpineJack® in maintaining vertebral height was stable over the three years, with only slight losses of anterior and central heights obtained initially, 4% at 12 months, and 6% at three years postoperatively. In addition, they reported that the implant surgery took on average significantly less time than kyphoplasty (23 minutes vs 32 minutes). There were no differences in terms of adverse effects, cement leakage, or adjacent fractures [56]. Another prospective multicenter study evaluated 108 patients with traumatic compression fractures treated with SpineJack® [57]. Two days after the interventions, the intensity of the pain had decreased by 81.5%, with an improvement of 91.3% in the Oswestry score and 21.1% in the EQ-VAS score at three months after the surgery. These results remained after one year of follow-up time. Local kyphosis significantly improved by 5.4° ±6.3 in the immediate postoperative period, with an improvement of 4.4° ±6.0 in comparison to the initial kyphosis maintained after one year of follow-up. After this period, there were 2.9% of fractures at adjacent levels and there were no problems related to the implants [57].

Baeesa et al. published a prospective study including 27 patients diagnosed with vertebral compression fracture of the thoracolumbar spine with both traumatic and osteoporotic origin treated with SpineJack® implants [43]. Pain measured by VAS score decreased from 7.0 preoperatively to 3.2 within 24 hours and remained at 2.2 at three-month, 2.1 at six-month, and 1.5 at 12-month follow-ups. The mean values show increased postoperative vertebral height compared to pre-intervention values in all nine measured areas. Mean height restorations, determined by three-dimensional CT were 3.56 mm for the anterior portion, 2.49 mm for central, and 1.28 mm for posterior, all maintained at the 12-month follow-up. The kyphotic angle was reduced from a preoperative value of 13.71° to 2.66°, the difference being statistically significant. Authors concluded that these implants allow for good clinical results in pain control and the possibility to reduce both vertebral kyphosis angles and fractured endplates, guaranteeing a more anatomical restoration of the whole vertebral body geometry (cortical ring and endplates) [43]. Similar outcomes were obtained by Noriega et al. in the same year and by Muñoz Montoya et al. three years later [58,59]. The SAKOS study, in a prospective, randomized, and multicentric study (141 patients from 13 hospitals in five countries), demonstrated the non-inferiority of SpineJack® implants after one year of follow-up, in vertebral reductions in osteoporotic fractures, compared to kyphoplasty [60]. There was a significantly greater increase in the intermediate height of the vertebral body in the SpineJack® group, compared to kyphoplasty, which corresponded to 1.14 ±2.61 mm vs 0.31 ±2.22 mm at six months and 1.31 ±2.58 mm vs 0.10 ±2.34 mm at 12 months postoperatively. However, no differences were noted in anterior and posterior heights, nor in segmental kyphosis. In addition, no differences were identified in terms of functional capacity and quality of life. Nevertheless, the improvement in pain was significantly higher in the SpineJack® group in the first month and at six months after surgery. The groups were similar in terms of cement leakage, all without clinical repercussions. However, significantly less cement was injected in the implants group (4.1 ±1.7 vs 5.9 ±2.3 cc), and this group also had significantly fewer fractures at adjacent levels (12.9% vs 27.3%) [60].

A prospective study by Kerschbaumer et al. followed, for an average of 2.3 years, 74 patients with 77 vertebrae with traumatic fractures [61]. In the first group of patients, SpineJack® implants were applied alone, while in the second group, SpineJack® was complemented with pedicle instrumentation in explosive fractures (A3) and with Magerl's B component. Regarding VAS, in group 1, it went from the initial 7.3 ±1.1 to 1.7 ±1.2 in the postoperative period and 0.7 ±1.6 at the end of the follow-up, while in group 2 these values were 7.1 ±0.8, 2 ±1.2, and 1.6 ±2.4, respectively. The final mean Oswestry score did not show any significant differences between groups, being 5.7 ±12.2 for group 1 and 11.6 ±13 for group 2. In the first group, the mean vertebral kyphosis angle increased from 13.3 ±6.1 preoperatively to 6.5˚ ±4.6 postoperatively, with a loss of reduction of 0.80.8 ±1.6 at the end of the follow-up. In turn, in group 2, the mean vertebral kyphosis angle increased from 15.3˚ ±5.7 preoperatively to 5.1˚ ±3.9 postoperatively, with a loss of reduction of 0.6˚ ±2 at the end of the follow-up. In the first group, the mean regional kyphosis angle increased from 8.3˚ ±7.2 preoperatively to 6.3˚ ±6.9 postoperatively, with a loss of reduction of 0.6˚ ±2.1 at the end of the follow-up. On the other hand, in group 2, the mean region kyphosis angle increased from 10.6˚ ±5.6 preoperatively to 2.9˚ ±4.7 postoperatively, with a loss of reduction of 1.8˚ ±4.5 at the end of the follow-up. The angulation improvements were significant in both groups, and the improvement in both vertebral (10.1˚ ±5 vs 6.8˚ ±4.9) and segmental (7.7˚ ±7.9 vs 2.6˚ ±3.7) kyphosis was significantly greater in the SpineJack® group associated with pedicle instrumentation, compared to that with SpineJack® alone, which the authors attribute to the fact that group 2 had a greater number of burst-type fractures (Magerl's A3) and, as such, vertebrae with more accentuated initial kyphosis, to the greater capacity to restore the height of the more comminuted vertebral body and to the additional indirect reduction with maneuvers with pedicle instrumentation. As for losses of reduction, these were slight and all occurred only up to three months after surgery, with the vertebral height remaining constant [61].

**Table 4:** Main current studies regarding the application of SpineJack® implants in the context of compression vertebral fractures VAS: visual analog scale; OSW: Oswestry Disability score; VA: vertebral body angle; SA: segmental angle, PMMA: polymethylmethacrylate

SpineJack® article	Study type	Fracture type	Number of fractured vertebrae	Mean follow-up period	Symptoms (VAS)	Function	Imaging	Complications	Conclusion
Vanni et al. (2012) [19]	Randomized comparative controlled trial, prospective (versus kyphoplasty)	A1 osteoporotic	150 in SpineJack® group	1 year	No difference in VAS scores between treatment groups	No difference in OSW scores between treatment groups	Vertebral height restoration >50% in 85% of cases	0	Spine Jack® is able to determine a safe vertebral body height restoration and ensure a gradual and controlled vertebral fracture reduction
Noriega et al. (2015) [57]	Randomized controlled clinical trial	Traumatic compression	108	1 year	VAS: 6.6 -> 1.4	OSW: 76.2 -> 14.2; EQ-VAS: 53.4 -> 71.5	VA improvement of 4.4°	2.9% adjacent fractures	The SpineJack® procedure is an effective, low-risk procedure for patients with traumatic VCF allowing a fast and sustained improvement in quality of life over 1 year after surgery
Noriega et al. (2015) [58]	Case series, prospective	Traumatic and osteoporotic compression (A1, A2, A3.1)	32	1 year	VAS: 6.8 -> 1.3	OSW: 65 -> 10.5; EQ-VAS: 36.2 -> 75.6	-	30.8% PMMA leakages	Promising results regarding safety and efficacy
Baeesa et al. (2015) [43]	Case series, prospective	Traumatic and osteoporotic compression	27	1 year	VAS: 7 -> 1.5		Mean height restorations: 3.56 mm for the anterior portion; 2.49 mm for central: 1.28 mm for posterior; VA: 12° -> 5.5°	7.4% adjacent fractures, 18.4% PMMA leakages	SpineJack® shows good clinical results in pain control and the possibility to reduce both vertebral kyphosis angles and fractured endplates
Muñoz Montoya et al. (2018) [59]	Case series, prospective	Traumatic and osteoporotic compression (A1, A2, A3, and A4)	20	6 months	VAS: 5.9 -> 3.1	OSW: 48.4 -> 26.8	-	-	An effective and safe way of handling vertebral compression fractures
Noriega et al.(2019) [56]	Randomized comparative controlled trial, prospective (versus kyphoplasty)	Osteoporotic compression	15 in SpineJack® group	3 years	VAS: 8 -> 1.4	OSW: 65.4 -> 6; EQVAS: 41 -> 75.1; final E-5D: 0.93	Anterior height improvement of 10%; central height improvement of 10%; VA improvement of 5°; SA improvement of 2.5°	6% height loss	Vertebral body height restoration/kyphosis correction was better with the SpineJack® procedure
Noriega et al.(2019) [60]	Randomized Controlled comparative trial (versus kyphoplasty)	Osteoporotic compression	68 in SpineJack® group	1 year	VAS: 7.8 -> 1.6	OSW: 65 -> 13.4; EQ-5D: 0.28 -> 0.83	Central height improvement of 1.31 mm	12.9% adjacent fractures	Study results demonstrated non-inferiority of the SpineJack® procedure to the predicate kyphoplasty. Radiographic superiority of the SpineJack® with regard to freedom from adjacent level fractures and minor superiority for midline vertebral height restoration
Kerschbaumer et al. (2019) [61]	Case series, prospective	Traumatic compression	77–63 in SpineJack group (SJ); 14 in SpineJack plus pedicular instrumentation group (SJ-inst)	3 years	SJ group: VAS: 7.3à0.7; SJ-inst group: VAS: 7.1à1.6	SJ group: final OSW: 5.7; SJ-inst group: final OSW: 11.6	SJ group: VA: 13.3°à7.3°; SA: 8.3°à6.9°; SJ-inst group: VA: 15.3°à5.7°; SA: 10.6°à4.7°	SJ group: 44% PMMA leakages; SJ-inst group: 50% PMMA leakages; 1 dura leak and 1 vertebral body collapse	The SpineJack® seems to be a promising tool in the treatment of traumatic vertebral fractures

One of the complications pointed out about vertebral body augmentation procedures is the occurrence of fractures at adjacent vertebral bodies as a result of overload due to increased rigidity of the operated level, in particular by the most frequently applied PMMA cement. Nevertheless, it remains controversial as to whether these fractures are due to the surgical procedure or if it is a natural evolution, for example, from an osteoporotic or tumoral disease reaching the spine [18,51,52,62-64]. Several authors argue that expandable intravertebral implants, in addition to restoring the vertebral body height and consequently eliminating excessive anterior overload and preventing domino effect, generally allow for a smaller volume of intrasomatic cement injection (demonstrations to be sufficient between 10-25% of vertebral body filling with cement, around 4.4 cc, on SpineJack® implants to ensure adequate stability and prevent vertebral collapse) compared to vertebroplasty and kyphoplasty, which is beneficial in not increasing a lot of the vertebral stiffness and also in decreasing extravasation rates and the risks associated with it [2,20,21,31,39,49,65].

## Conclusions

Current scientific evidence regarding the use of expandable intravertebral implants in thoracolumbar vertebral fractures shows favorable radiographic and functional outcomes. The type of fractures that benefit most from this intervention and the exact indications for the use of these implants in traumatology remain topics under discussion; however, the percutaneous transpedicular access, the ability to anatomically reconstruct the vertebral body, particularly the endplates and the ability to maintain their height, make expandable intravertebral implants an attractive option in the treatment of compression fractures of the vertebral bodies. However, more prospective, randomized, and large-scale blinded studies are still needed, especially comparative studies between treatments and about the preferential use of an expansive implant over others, in order to definitively establish the effectiveness and indications of each of these devices.
